# Reduction in parvalbumin-positive interneurons and inhibitory input in the cortex of mice with experimental autoimmune encephalomyelitis

**DOI:** 10.1007/s00221-014-3944-7

**Published:** 2014-04-26

**Authors:** Anna Falco, Roberta Pennucci, Elena Brambilla, Ivan de Curtis

**Affiliations:** 1 Cell Adhesion Unit, Division of Neuroscience, San Raffaele Scientific Institute, via Olgettina, 58-20132 Milan, Italy; 2 Neuroimmunology Unit, Division of Neuroscience, San Raffaele Scientific Institute, via Olgettina, 58-20132 Milan, Italy; 3 Università Vita-Salute San Raffaele, via Olgettina, 58-20132 Milan, Italy

**Keywords:** Experimental autoimmune encephalomyelitis, Inhibitory synapses, Interneurons, Parvalbumin

## Abstract

In multiple sclerosis (MS), inflammation leads to damage of central nervous system myelin and axons. Previous studies have postulated impaired GABA transmission in MS, and recent postmortem analysis has shown that GABAergic parvalbumin (PV)-positive interneurons are decreased in the primary motor cortex (M1) of patients with MS. In this report, we present evidence for the loss of a specific population of GABAergic interneurons in the experimental autoimmune encephalomyelitis mouse model of MS. Using experimental autoimmune encephalomyelitis, we evaluated the distribution of both PV-positive interneurons and of the inhibitory presynaptic input in the M1 of experimental autoimmune encephalomyelitis and control mice. Our results demonstrate a specific decrease in the number of PV-positive interneurons in the M1 of mice with experimental autoimmune encephalomyelitis. We detected a significant reduction in the number of PV-positive interneurons in the layers II and III of the M1 of diseased mice, while there was no difference in the number of calretinin (CR)-positive cells between animals with experimental autoimmune encephalomyelitis and control animals. Moreover, we observed a significant reduction in the inhibitory presynaptic input in the M1 of treated mice. These changes were specific for the mice with elevated clinical score, while they were not detectable in the mice with low clinical score. Our results support the hypothesis that reinforcing the action of the GABAergic network may represent a therapeutic alternative to limit the progression of the neuronal damage in MS patients.

## Introduction

Loss of neuronal function is an important pathological feature of multiple sclerosis (MS), and there is evidence for the contribution of neuronal damage toward clinical disability (Chard et al. 2002). Analysis on postmortem control and MS human brains has shown a reduction in the expression of a number of genes important in the neurotransmission by GABAergic inhibitory neurons in the motor cortex (M1) of MS patients (Dutta et al. 2006). GABAergic interneurons may be grouped on the basis of the expression of different calcium binding proteins, such as parvalbumin (PV) and calretinin (CR) (DeFelipe 1997; Grateron et al. 2003; Cotter et al. 2002). PV and CR are members of the superfamily of EF-hand calcium binding proteins, which have a role in buffering the intracellular calcium (Baldellon et al. 1998). Defects in the expression of these calcium binding proteins have been implicated in a number of neurological pathologies including MS (Beers et al. 2001; Eyels et al. 2002). In particular, the data on postmortem MS brains have indicated a reduction in the expression of the PV gene as well as the reduced extension of neurites in PV-expressing interneurons within normal appearing gray matter in MS patients (Dutta et al. 2006). A comparative confocal microscopy analysis on the distribution of the immunoreactivity for different calcium binding proteins from normal and MS postmortem brains has shown a specific reduction in the number of PV-positive interneurons within layer II of the human MS M1, while no changes could be detected in the number of CR-positive interneurons (Clements et al. 2008).

Given the decrease in PV-positive interneurons in the brain of MS patients, we tested if a similar phenotype could be induced in an animal model to study MS. We looked at the effects of experimental autoimmune encephalomyelitis (EAE) on the number and differentiation of PV-positive interneurons in the M1 of wild-type mice by comparing treated (EAE) and control animals, to establish whether we could reproduce the defect observed in the PV-positive cortical population of MS patients by using a well-established mouse model for EAE (Pluchino et al. 2003). We found that the number of PV-positive cells as well as the density of inhibitory presynaptic inputs on cortical pyramidal cells is markedly and specifically reduced in the M1 of EAE mice.

## Materials and methods

### Induction of EAE

EAE was induced according to published procedures (Furlan et al. 2009). Briefly, C57BL/6 female mice were injected at the same age (8 weeks, with a starting weight of 20–22 g). The mice were immunized with the same amount of immunogen by subcutaneous injection with 300 μl of MOG (35–55) peptide (200 μg, Multiple Peptide System) in incomplete Freund’s adjuvant containing 8 mg/ml of Mycobacterium tuberculosis (strain H37Ra; Difco). Pertussis toxin (500 ng; from Sigma) was injected intravenously on the day of the immunization and 2 days later. Body weight and clinical score were recorded daily. The following clinical scores were assigned: 0 = healthy; 1 = limp tail; 2 = ataxia and/or paresis of hind limbs; 4 = tetra paralysis; 5 = moribund or death. All procedures involving animals were performed according to the guidelines of the Animal Ethical Committee of our Institute.

### Antibodies

Antibodies used in this study were as follows: rabbit polyclonal antibodies against CR (Chemicon International), PV (PV-28, Swant), vesicular GABA transporter (vGAT, Synaptic Systems), and glial fibrillary acidic protein (GFAP, Dako Cytomation); goat polyclonal antibody against PV (PVG-214, Swant). The rat monoclonal antibody against CD45 was a kind gift of Gabriela Constantin (University of Verona). Primary antibodies were detected by Alexa Fluor-conjugated secondary antibodies (Invitrogen) or biotinylated rabbit anti-rat and goat anti-rabbit immunoglobulins (Vector Laboratories).

### Immunohistochemistry and immunofluorescence

Mice were sacrificed 30 days after immunization. Mice were fixed under deep anesthesia by transcardial perfusion with 4 % paraformaldehyde in phosphate-buffered saline (PBS). The brain was removed and postfixed overnight at 4 °C. For immunostaining, samples were washed with PBS, cryoprotected with sucrose in PBS, and frozen in optimal cutting temperature compound (VWR International Ltd.). 12-to 20-μm-thick sections were stained with cresyl violet or used for immunostaining. For immunohistochemistry, sections were incubated with primary antibodies overnight at 4 °C. Primary antibodies were detected with the Vectastain Elite ABC Kit (Vector Laboratories). Sections were viewed with a Zeiss Axioplan2 microscope with AxioCam MRc5 digital camera (Carl Zeiss MicroImaging). For immunofluorescence, primary antibodies were revealed by incubation for 1.5–2 h with Alexa Fluor-conjugated secondary antibodies and 4′,6-Diamidino-2-Phenylindole (DAPI, Sigma-Aldrich) for nuclear staining. Sections were viewed with a Zeiss Axiovert 135 TV equipped with a QImaging Exi-Blue camera (Carl Zeiss MicroImaging). Confocal analysis was performed with a Leica TCS SP2 (Leica Microsystems). Quantification with ImageJ software (NIH, Bethesda, MD) was performed on 6–7 sections from at least 3 different mice per genotype for each experimental condition.

### Apoptosis

Terminal deoxynucleotidyl transferase dUTP nick end labeling (TUNEL) was performed using the DeadEnd Colorimetric TUNEL System Kit (Promega). Sections including the M1 were processed following the manufacturer’s instructions and analyzed as described (Pennucci et al. 2011). Sections of the interdigit region of E13.5 wild-type mice (not shown) were used as positive controls (Fernandez-Teran et al. 2006).

### Quantitative analysis

PV-positive and CR-positive cells were quantified in 12–15 parasagittal sections from 3 mice per experimental condition. The density of PV-positive cells was evaluated from similar areas of the M1 using the ImageJ software. For quantification of the presynaptic GABAergic terminals, 24–26 fields from sections of the M1 of 3 mice per experimental condition were used. The fluorescent signal of PV and vGAT was evaluated in equal areas from parasagittal sections including comparable levels of layers II–III of the M1 of EAE and control mice. Laser power and settings were the same for all samples within the same experiment. Images were analyzed with ImageJ. The area occupied by fluorescent signals above the background and the mean gray value were quantified in layers II–III of the M1. The mean gray value was calculated as the average intensity of fluorescence per unit area. Quantitative analysis of the colocalization of PV with vGAT was performed using ImageJ.

## Results

### EAE causes a reduction in the number of PV-positive interneurons in the primary motor cortex

**Fig. 1:**
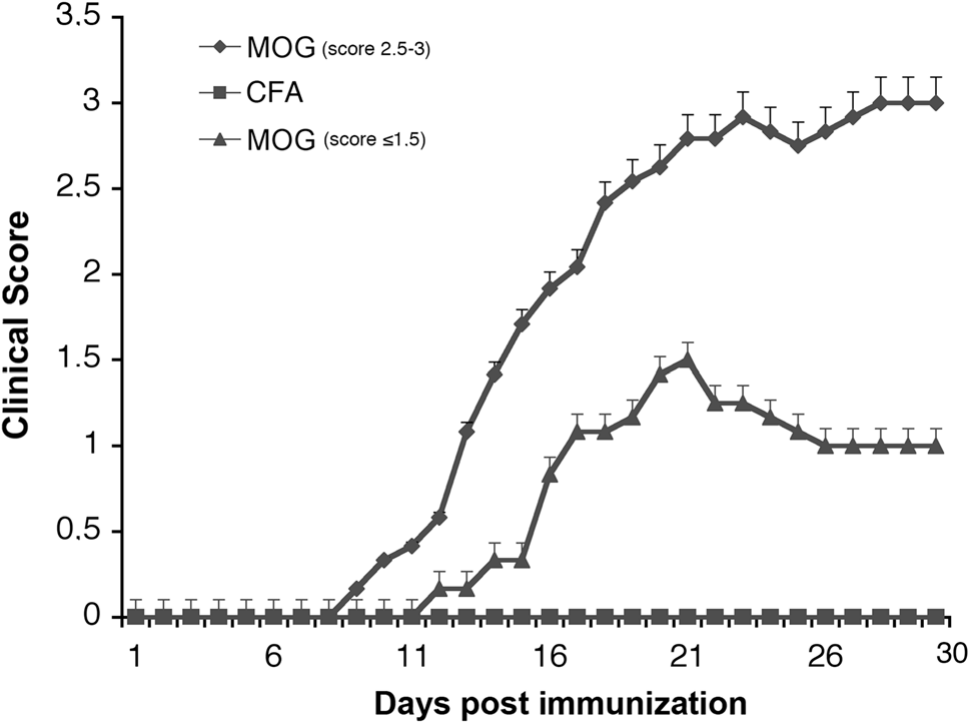
EAE clinical score. A group of treated mice (MOG, score 2.5–3, *n* = 5) displays a pronounced clinical score compared with control mice injected with Freund’s adjuvant only (CFA, *n* = 10), while a second group of mice (MOG, score ≤1.5, *n* = 4) displays a lower clinical score. Data in the graph are expressed as mean ± SEM

**Fig. 2:**
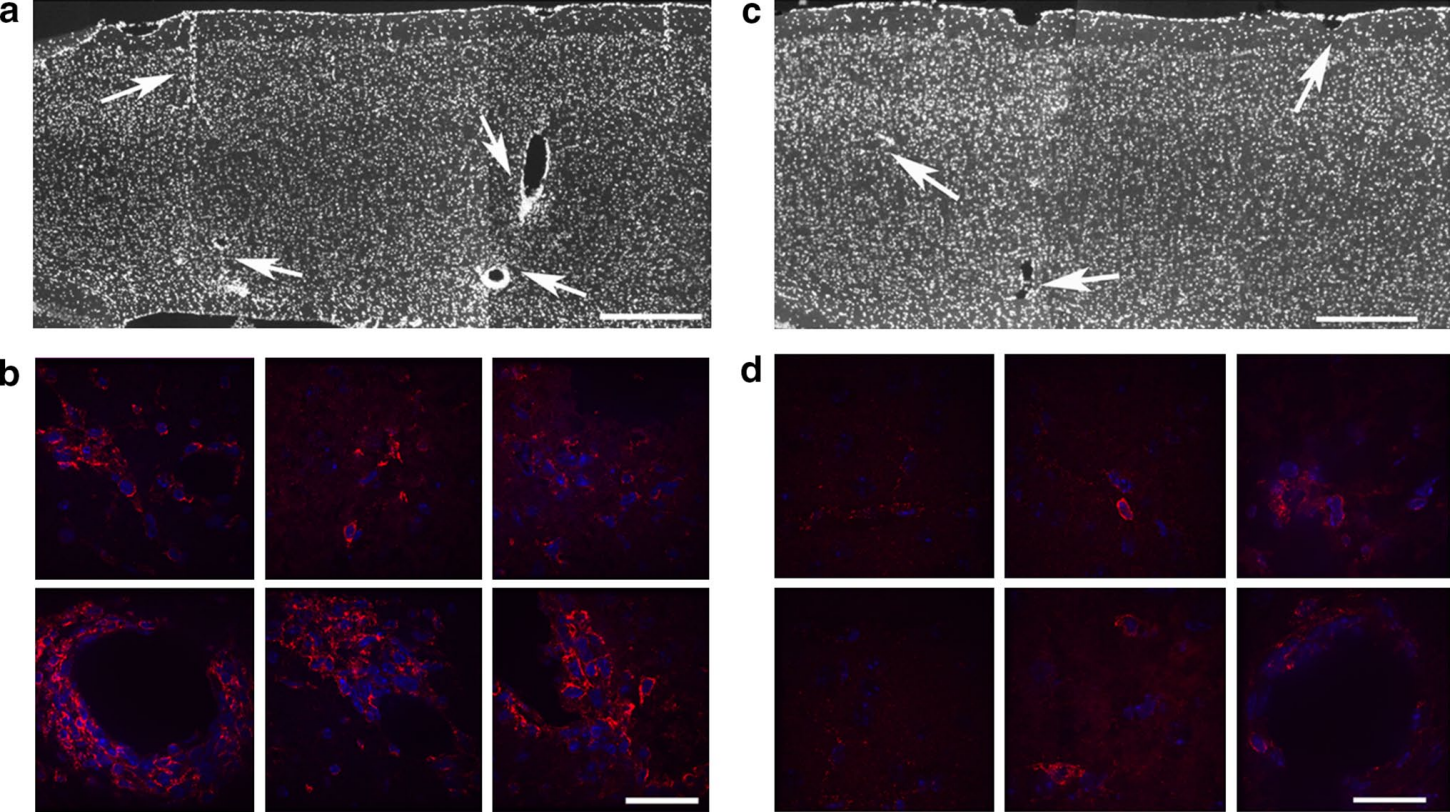
The brain of EAE animals shows infiltration by leukocytes. Sections were immunostained for CD45 (*red*) and DAPI (*blue*) to identify inflammatory infiltrates of leukocytes. **a** Low magnification of the motor cortex of a mouse with high clinical score. *Arrows* indicate some inflammatory lesions. *Scale bar*, 200 μm. **b** Higher magnification of the inflammatory lesions in the primary motor cortex shows in **a**. *Scale bar*, 20 μm. **c** Lower magnification of the motor cortex of a mouse with low clinical score. *Arrows* indicate some inflammatory lesions. *Scale bar*, 200 μm. **d** Higher magnification of inflammatory lesions in the primary motor cortex shows in **c**. *Scale bar*, 20 μm

**Fig. 3:**
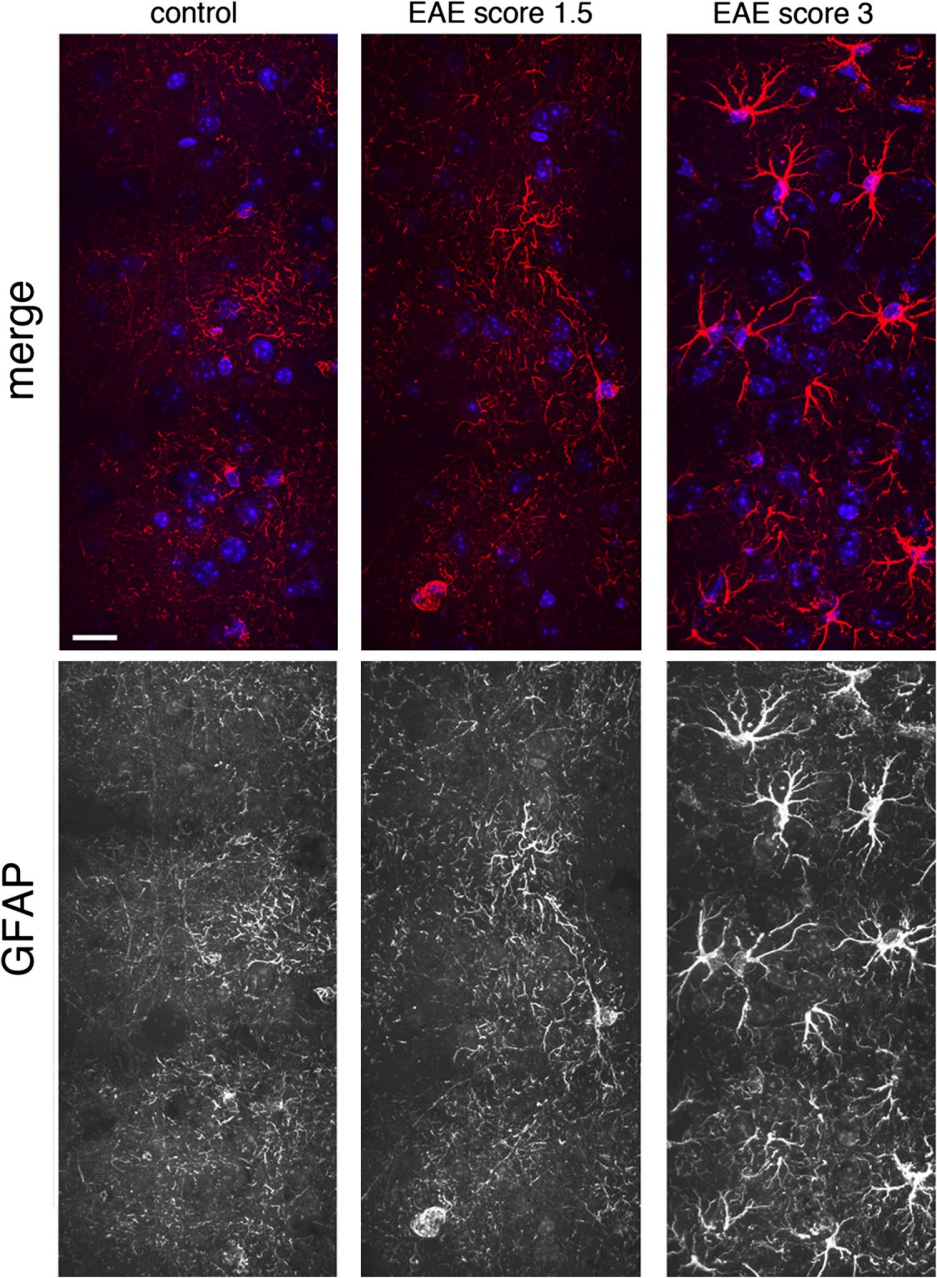
Increased number of astrocytes in the cortex of EAE mice. Z-stack projections of confocal images of layers II–III of the M1 of EAE mice immunostained for GFAP (*red*) and DAPI (*blue*). *Scale bar*: 20 μm. The signal for GFAP is stronger in the motor cortex of EAE mice with pronounced clinical score (*right*). The signal for GFAP is very low in the motor cortex of control mice (*left*) and of EAE mice with low clinical score (*center*)

We examined para sagittal sections of primary M1 of EAE mice with elevated (2.5–3) and lower (≤1.5) clinical score (Fig. 1) by immunostaining with antibodies for CD45 to detect the presence of infiltrating leukocytes. We found several CD45-positive cells in the cortex of EAE mice with high clinical score. In mice with elevated clinical score (2.5–3), the signal for CD45 was clearly more extended with respect to the signal observed in mice with low score (Fig. 2). The immunohistological analysis showed that lesions in the cortex of the animals with elevated clinical score were surrounded by more numerous clusters of CD45-positive leukocytes when compared to lesion found in less affected mice. We thus confirmed the presence of inflammatory lesions in the subpial and across the cortical layers, as previously described (Mangiardi et al. 2011). We also immunostained brain sections to detect GFAP as a marker of astrocytes, which were increased in the cortex of EAE mice with elevated clinical score (2.5–3) with respect to control mice or EAE animals with lower scores (Fig. 3). These results confirm that gliosis is present in the cortex of EAE mice with elevated clinical score.

**Fig. 4:**
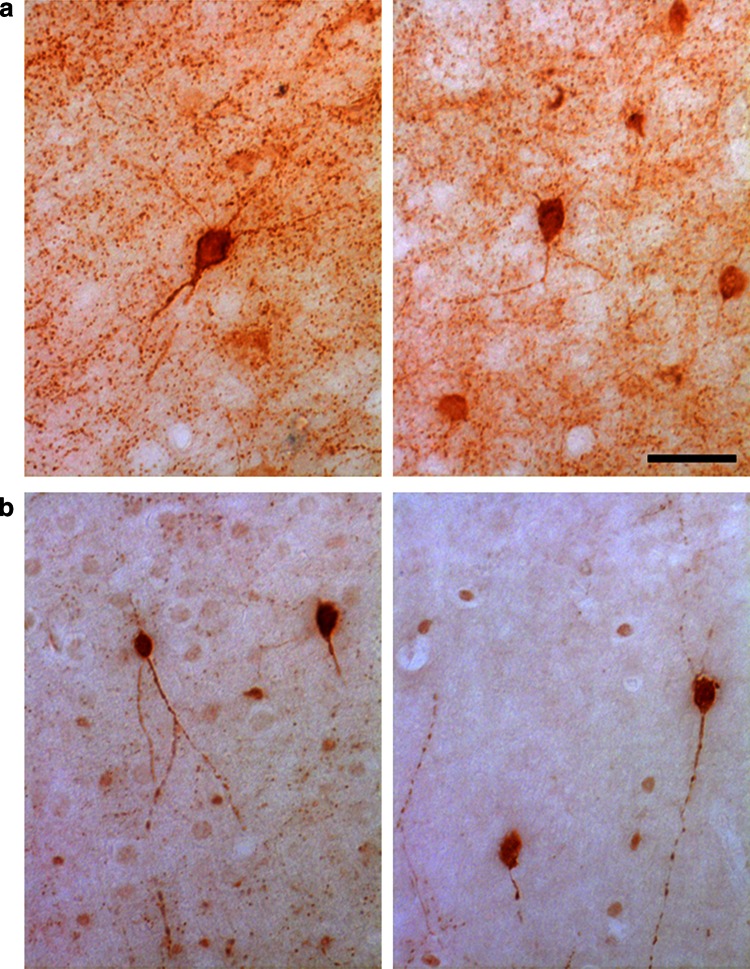
PV-positive **a** and CR-positive **b** GABAergic interneurons in the M1 of control mice

**Fig. 5:**
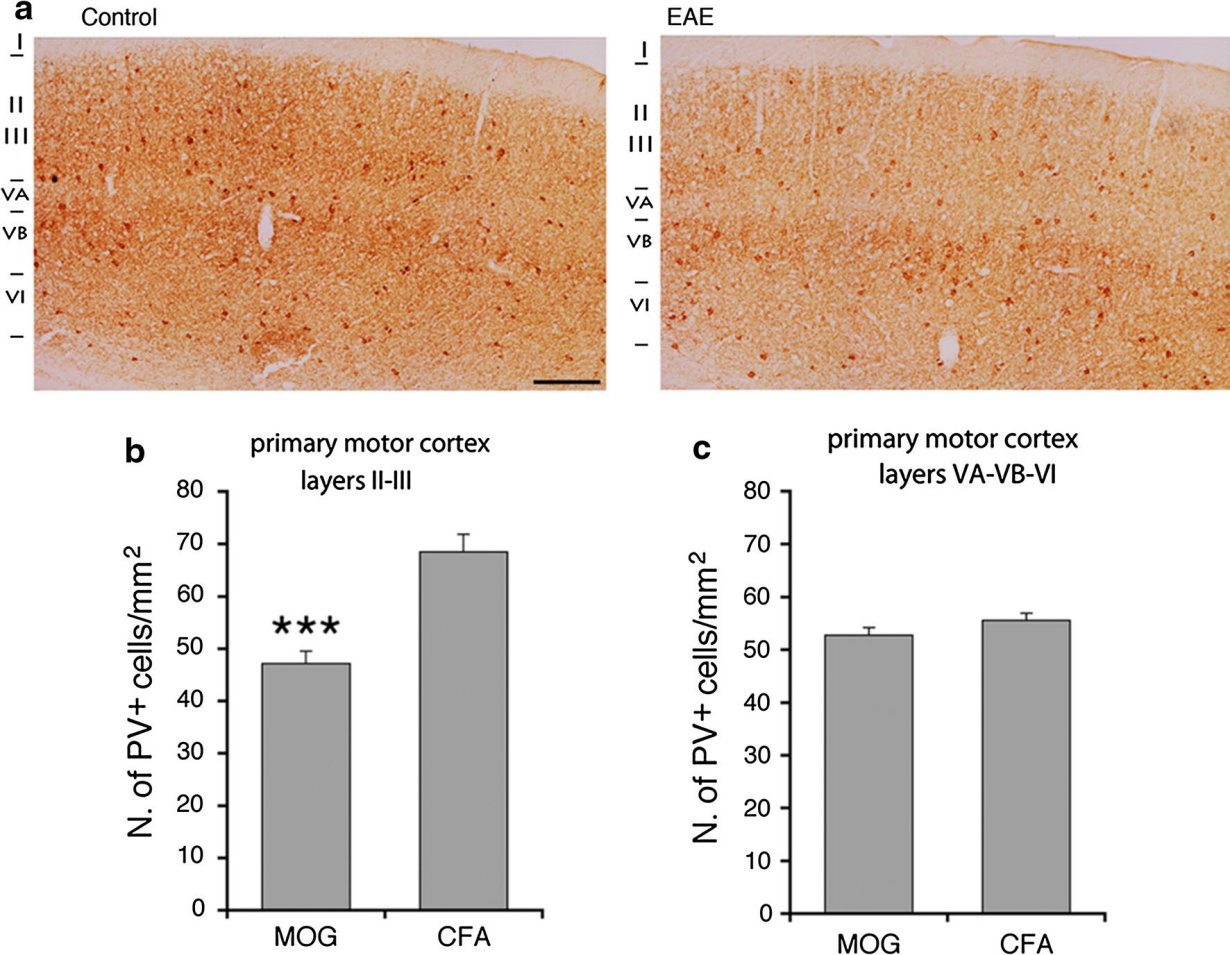
EAE causes the reduction in PV-positive interneurons in layers II–III of the M1. **a** PV staining of the M1 of EAE mice with high clinical score (*right*) and control mice (*left*). *Scale bar*, 200 μm. **b** Number of PV-positive neurons/mm^2^ in layers II–III of the M1 from EAE mice with elevated clinical score and control mice. **c** Number of PV-positive neurons/mm^2^ in layers V–VI of the M1 from EAE mice with pronounced clinical score and control mice. *Graph bars* are normalized means ± SEM (*n* = 30 sections from 5 mice/experimental condition). ***P < 0.001

**Fig. 6:**
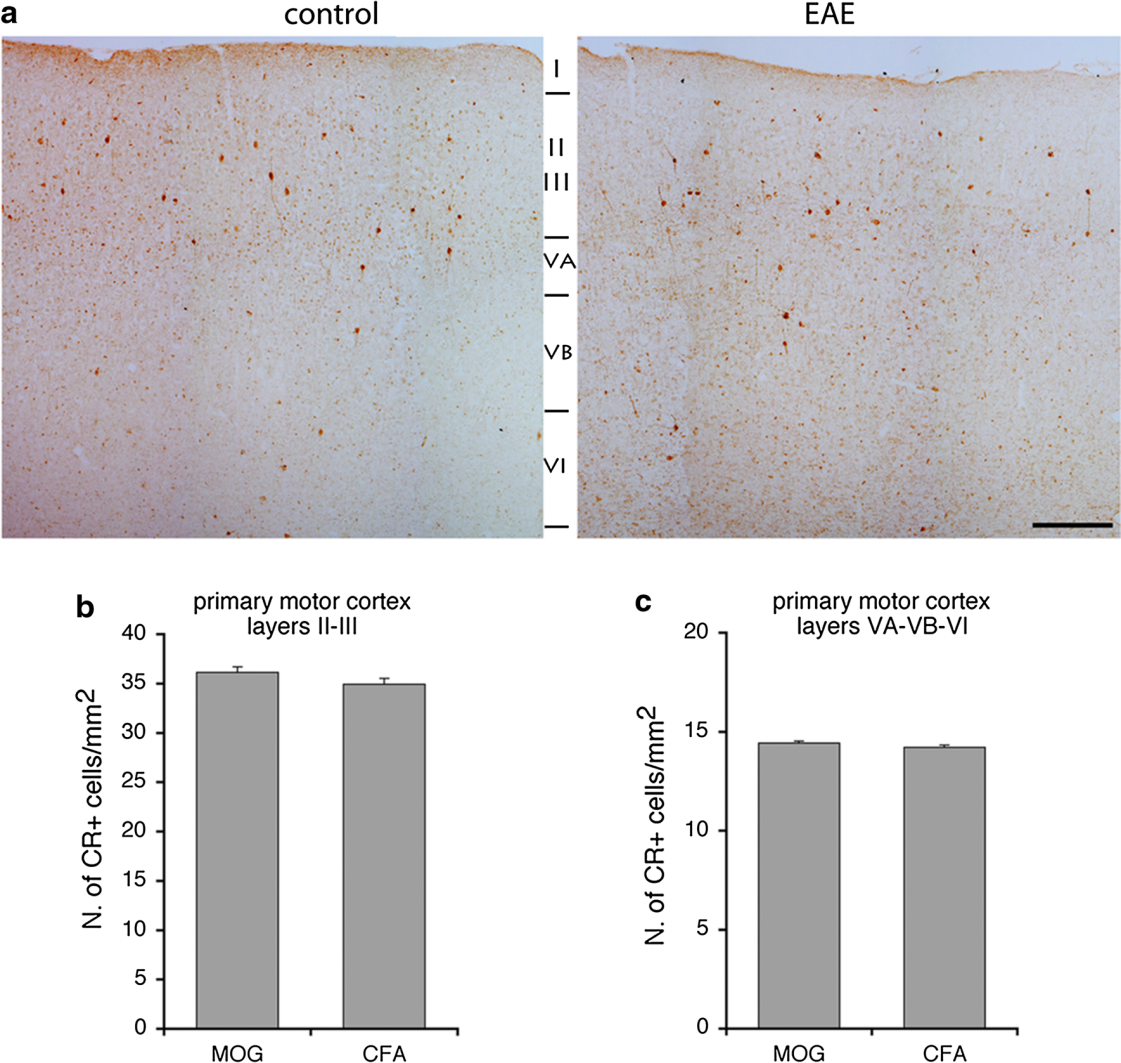
EAE does not affect the number of CR-positive cells in the M1. **a** CR staining of the M1 of EAE and control mice. *Scale bar*, 200 μm. **b** Number of CR-positive neurons/mm^2^ in layers II–III of the M1 of EAE and control mice. **c** Number of CR-positive neurons/mm^2^ in layers V–VI of the M1 from EAE and control mice. *Graph bars* are normalized mean ± SEM (*n* = 24 sections from 5 mice/experimental condition)

**Fig. 7:**
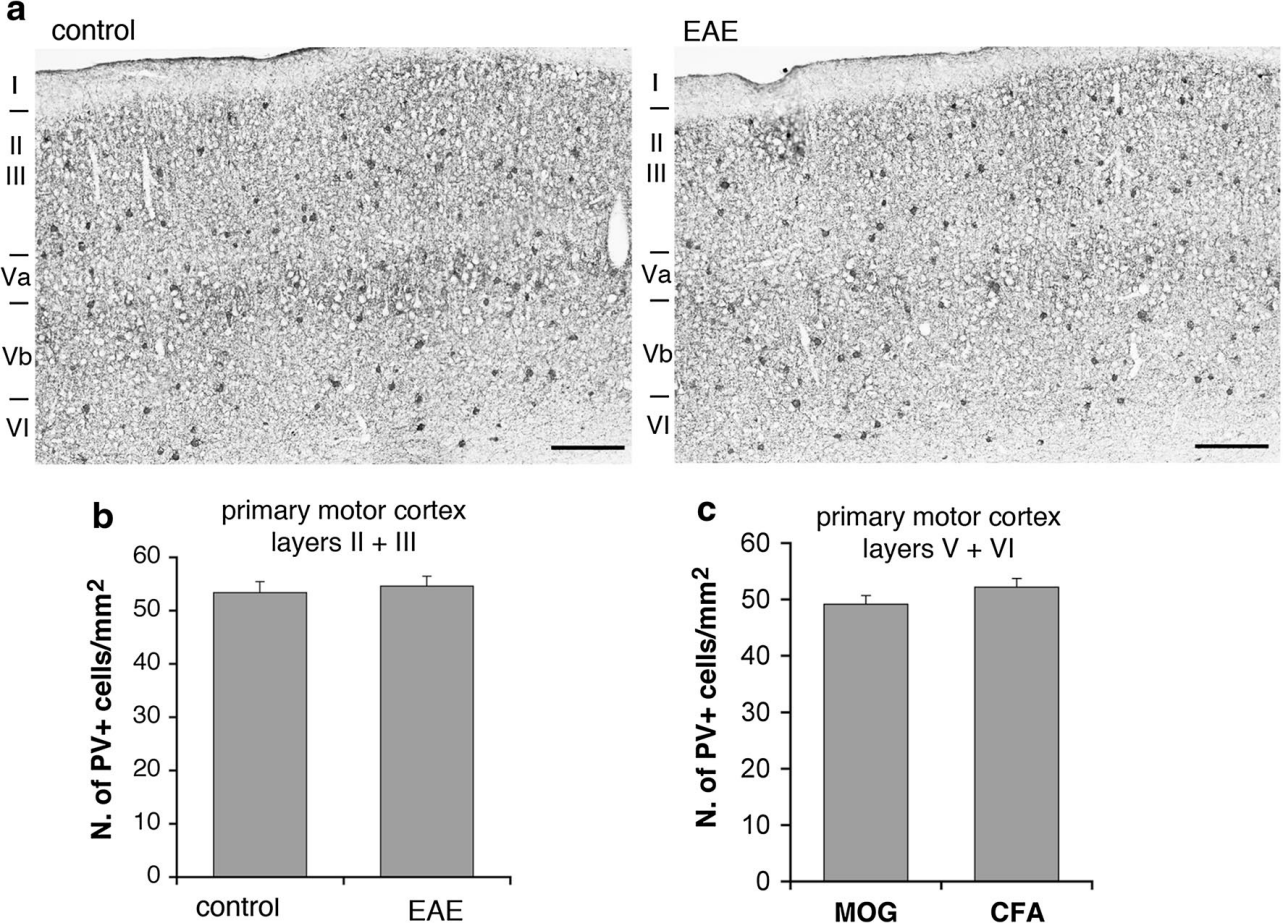
PV-positive cells are not affected in the cortex of EAE mice with low clinical score **a**. *Scale bars*, 200 μm. **b** Number of PV-positive neurons/mm^2^ in layers II–III of the M1 from EAE mice with low clinical score and control mice. **c** Number of PV-positive neurons/mm^2^ in layers V–VI of the M1 from EAE mice with low clinical score and control mice. *Graph bars* are normalized mean ± SEM (*n* = 7–15 sections from 3 mice/experimental condition)

PV-positive GABAergic interneurons are reduced in the striatum (Rossi et al. 2011) and in the hippocampal CA1 area (Ziehn et al. 2010) of EAE mice. Moreover, it has been documented a significant reduction in the number of PV-positive interneurons within layer II of the M1 of patients with MS, while no concurrent change in the number of CR-positive neurons was observed (Clements et al. 2008). We utilized immunohistochemistry to determine whether PV-positive interneurons were affected in the M1 of EAE mice. We sacrificed mice with pronounced (2.5–3) and low clinical scores (≤1.5) 30 days after immunization, to look at PV- and CR-positive interneurons in the M1. Cortical PV-positive cells (Fig. 4a) may include large basket and chandelier type morphologies, while CR-positive cells (Fig. 4b) may include Cajal–Retzius, double bouquet, and bipolar morphologies (Amitai et al. 2002). In EAE mice with pronounced clinical score, the number of PV-positive interneurons in the M1 was reduced. A 30 % reduction was restricted to layers II–III, whereas no differences were detected in the layers V–VI (Fig. 5). In contrast to PV-positive cells, there was no difference in the number of CR-positive cells between EAE and control mice in the same cortical areas (Fig. 6). We performed the same analysis in the M1 of EAE mice with low clinical score (≤1.5). Our data show no differences in the number of PV-positive cells in the M1 of these mice (Fig. 7).

**Fig. 8:**
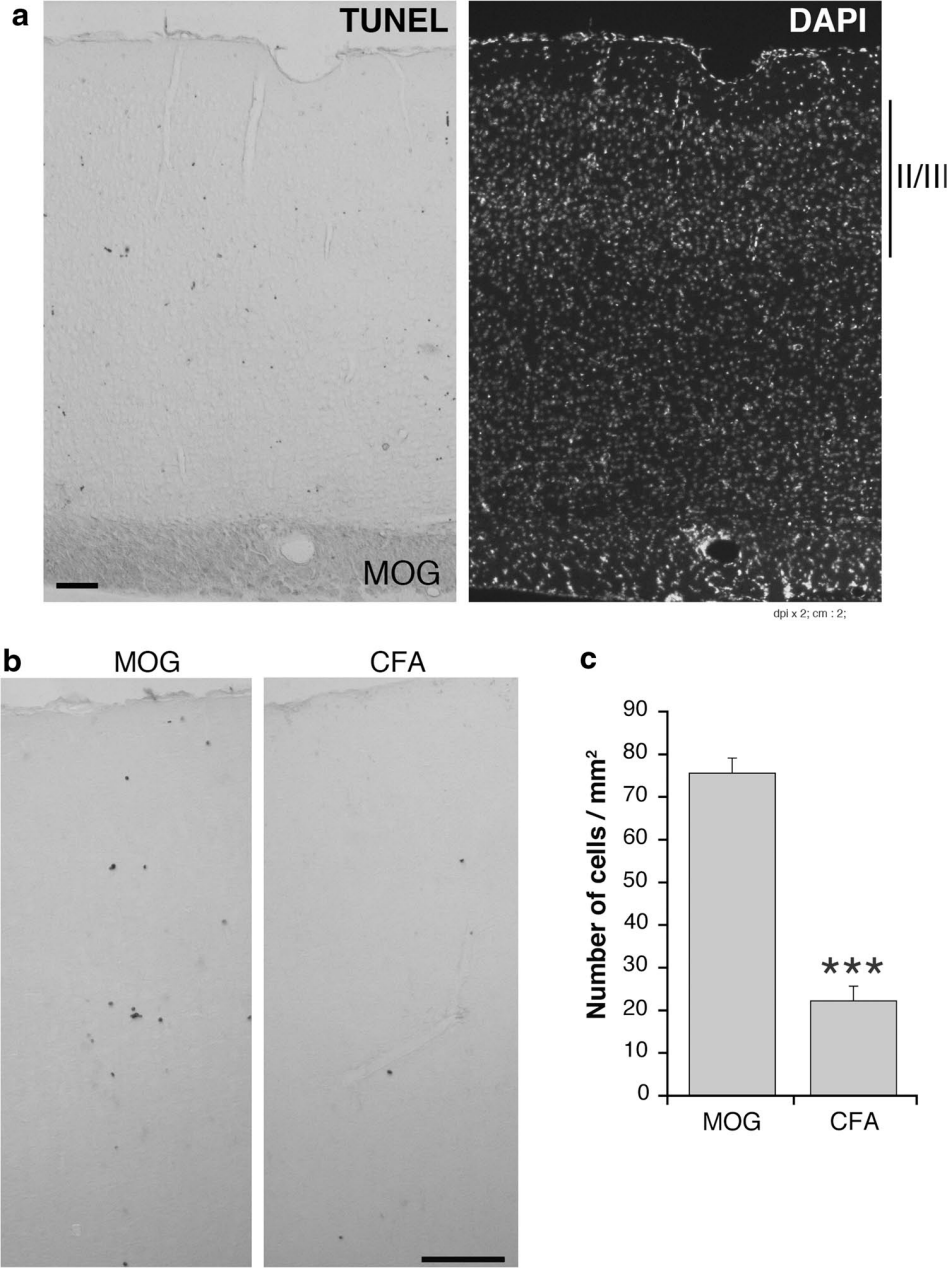
Analysis of cell death in the M1. **a,b** TUNEL staining of the motor cortex from EAE mice with high clinical score (**a** and **b**, *left panels*) and control (**b** *right panel*) mice. *Scale bars*, 100 μm. **c** Number of TUNEL-positive cells/mm^2^ in layers II–III of the M1 from EAE mice with high clinical score and control mice. *Graph bars* are mean ± SEM (*n* = 15 sections from 3 mice/experimental condition). ***P < 0.001

The loss of PV-positive cells observed in the M1 of EAE mice could be due to the neuronal death associated to the development of the disease (Mangiardi et al. 2011). We could confirm a significant increase in cell death in the M1 of EAE mice with high clinical score, with respect to control mice, as detected by TUNEL staining (Fig. 8). These data suggest that the loss of the PV-positive interneurons in the M1 of EAE mice may be due to the death of these neurons.

### The reduction in PV-positive interneurons in the primary motor cortex corresponds to a reduced inhibitory presynaptic input

**Fig. 9:**
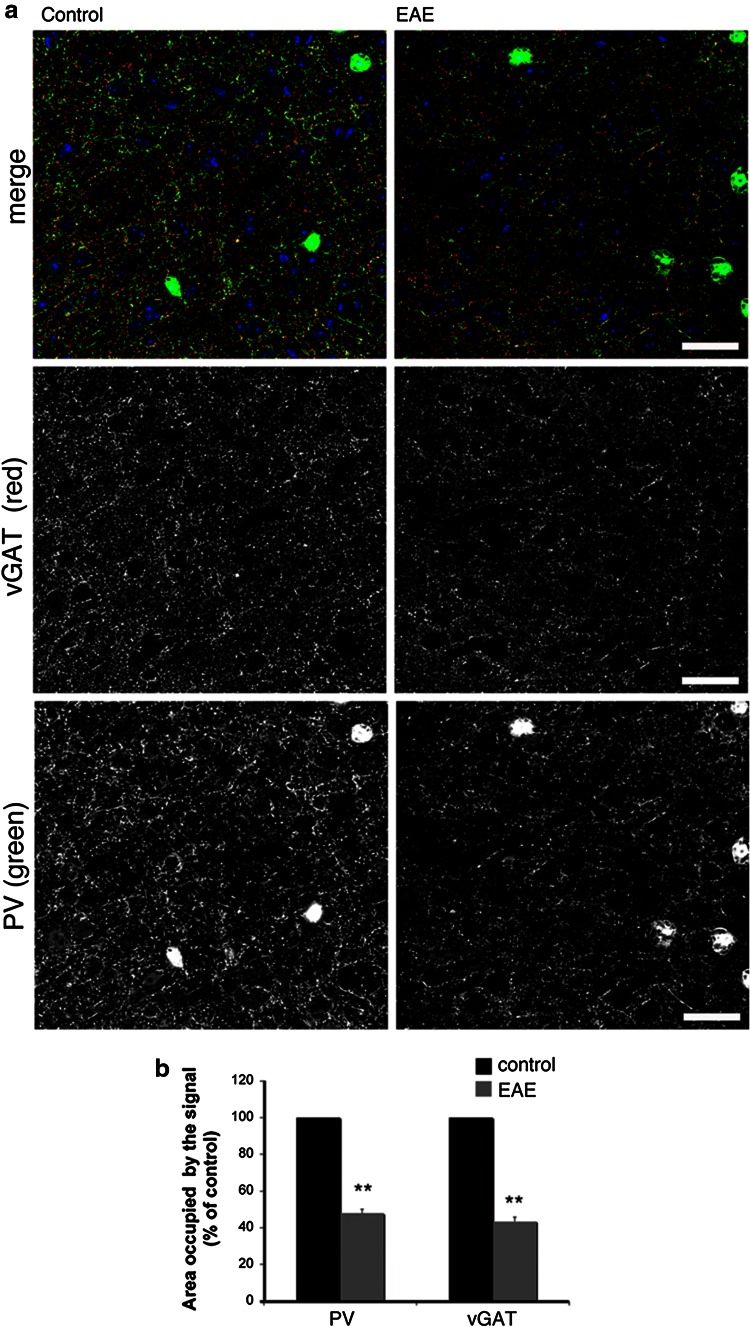
The inhibitory presynaptic input is reduced in the M1 of EAE mice. **a** Confocal images of layers II–III of the M1 immunostained for the presynaptic marker vGAT (*red*), PV (*green*) and DAPI (*blue*). *Scale bars*, 50 μm. **b** Reduction of the signal for both PV and vGAT in layers II–III of the M1 of EAE and control mice. *Graph bars* are normalized mean ± SEM (*n* = 26 cortical fields from 3 mice/experimental condition). **P < 0.005

**Fig. 10:**
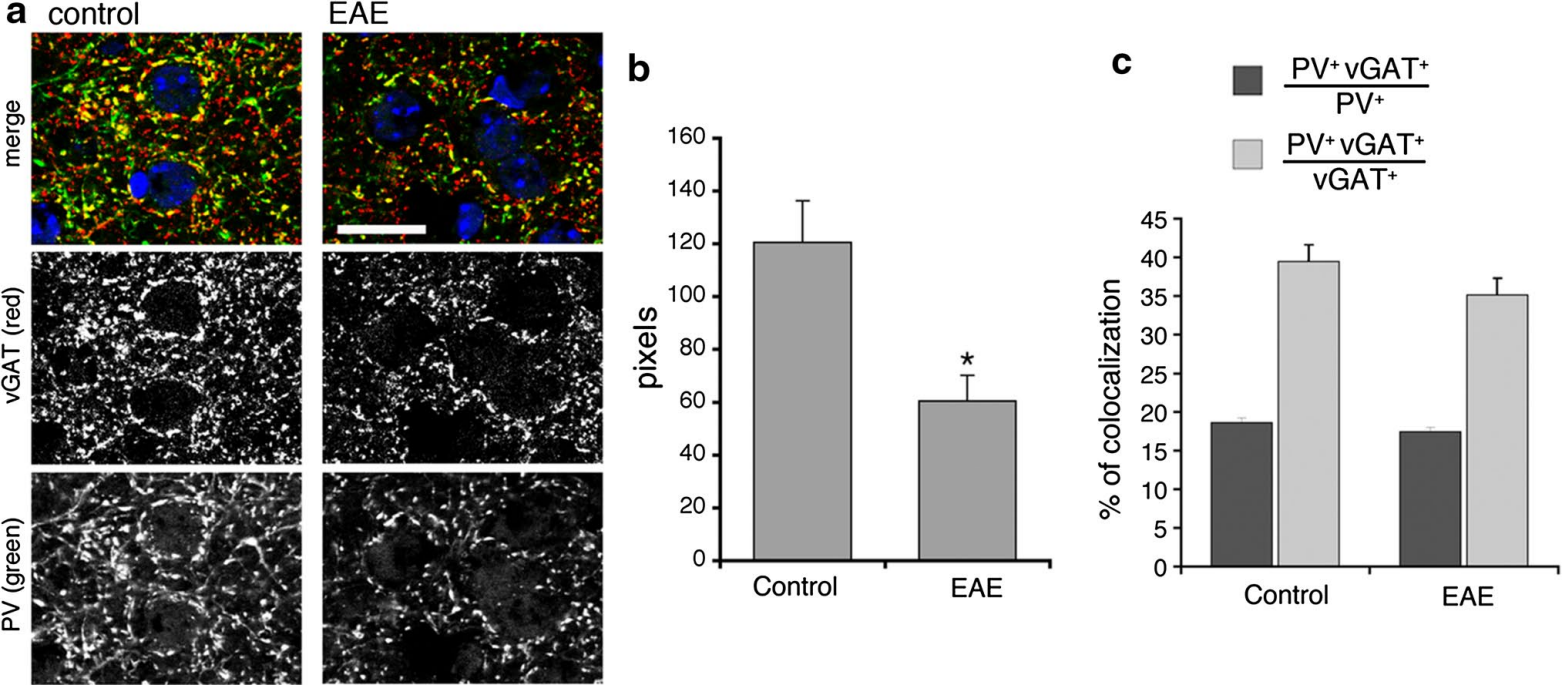
Colocalization of PV with GABAergic presynaptic terminals in the M1 of EAE and control mice. **a** Confocal images from layers II–III of the M1 immunostained for the presynaptic marker vGAT (*red*), PV (*green*), and DAPI (*blue*). *Scale bar*, 20 μm. **b** Area of colocalization of PV with vGAT in the cortex of EAE and control mice. **c** Percentage of the area of colocalization of vGAT and PV with respect to either the total area positive for vGAT (*light gray*) or the total area positive for PV (*dark gray*). *Graph bars* in **b** and **c** are mean ± SEM (*n* = 9 from 3 mice/experimental condition)

Previous studies have postulated impaired GABA transmission in MS and in EAE (Dutta et al. 2006; Rossi et al. 2011). A significant reduction in the number of PV-positive striatal cells accompanied impaired GABA signaling detected by electrophysiological, morphological, and biochemical analysis (Rossi et al. 2011). Moreover, it has been demonstrated a reduction in the transcripts for genes related to the GABAergic phenotype and a decrease in the density of inhibitory interneuron processes in the M1 samples from MS patients (Dutta et al. 2006). To assess if the reduction in PV-positive interneurons observed in the present study was accompanied by an effect on the inhibitory input in mice with EAE, we analyzed markers of GABA transmission by immunofluorescence confocal analysis. This analysis has shown that the PV-positive processes, as well as the vGAT-positive GABAergic presynaptic terminals were reduced in the M1 (layers II–III) of EAE mice with high score for the disease (2.5–3) (Fig. 9a). We quantified the percentage of cortical area positive for either PV or vGAT in sections from control (CFA, Freund’s adjuvant only) and EAE M1. The area occupied by either marker was strongly reduced in the EAE mice with high score with respect to control animals: the area occupied by PV was reduced by 52.2 %, while the area occupied by vGAT was reduced by 56.5 % (Fig. 9b). Interestingly, quantification in the M1 showed that the extension of colocalization of PV with vGAT at the inhibitory presynaptic sites was also decreased in the EAE mice compared to control animals (Fig. 10a,b). Therefore, EAE induced a decrease in the presynaptic sites formed by PV-positive interneurons. On the other hand, the fraction of PV-positive structures positive for vGAT, as well as the fraction of vGAT-positive structures positive for PV were similar between EAE and control mice (Fig. 10c), suggesting that the input by other types of GABAergic interneurons may also be affected in the EAE mice. Overall, our data indicate that the reduction in the number of PV-positive interneurons in the M1 of EAE mice corresponds to a reduction in the inhibitory presynaptic input on principal cortical neurons.

## Discussion

In this study, we have addressed the effects of EAE on the cortical GABAergic inhibitory network. Based on previous findings in MS patients (Clements et al. 2008), we have considered two classes of interneurons present in the M1: the PV-positive and the CR-positive cells. Consistently with the findings in the human MS patients, we observed a specific negative effect of EAE on the PV-positive interneurons that appear to be more sensitive to the disease when compared to the CR-positive inhibitory interneurons.

The calcium binding proteins PV and CR are expressed in distinct GABAergic interneuron populations, and they are known to buffer calcium concentrations to protect neurons from rises in intracellular calcium (Baldellon et al. 1998). Perturbations in calcium binding proteins have been suggested to accompany a number of pathological conditions including MS (Beers et al. 2001; Eyels et al. 2002). These two interneuron populations are characterized by different developmental, electrophysiological, and structural parameters (Amitai et al. 2002; Grateron et al. 2003; DeFelipe 1997). The current study reveals a significant loss of PV-positive interneurons in the M1 of EAE mice when compared to controls. This change was not associated to any alteration in the number of CR-positive interneurons, suggesting a specific sensitivity of the PV-positive cells to the disease. The reduction in PV-positive interneurons was restricted to cortical layers II–III, whereas no differences were detected in layers V–VI. Furthermore, these changes were evident only when high clinical scores were reached, since no differences in the number of PV-positive interneurons could be observed in the M1 of mice with low clinical score. These data suggest that the loss of cortical PV-positive interneurons may represent a relatively late hallmark of the disease.

Previous studies have indicated that the presynaptic axon terminals of PV-positive cells innervate either the axon initial segment or the cell body and proximal dendrite of pyramidal neurons in layers III of the frontal cortex (Lewis et al. 2012). We have quantified the signals for PV and for the GABAergic presynaptic marker vGAT (Tafoya et al. 2006; Centonze et al. 2008) to assess if the observed reduction in PV-positive cells corresponds to a lower inhibitory input in the cortex. The analysis showed that the reduction in the GABAergic presynaptic terminals in the M1 of EAE mice was evident only in animals with a high score for the disease.

In conclusion, our data demonstrate that the inhibitory circuitry of the M1 is affected by EAE and suggest that the population of PV-positive interneurons is specifically vulnerable to the pathological insult that is subsequent to EAE progression. The reason for this specific vulnerability is unknown. Since the effects on PV-positive neurons were only detectable in mice with elevated clinical score, the results suggest that loss of these neurons is a hallmark of the advanced stages of the disease. Finally, together with previous studies implicating a GABAergic defect in other areas of the brain during the development of the disease (Rossi et al. 2011; Ziehn et al. 2010), our results support the hypothesis that the reinforcement of the signaling linked to the action of GABAergic interneurons may be a valid therapeutic alternative to control the progression of the neuronal damage in MS patients.
